# Psychological resilience and disaster preparedness among parents of children with hearing impairments: a cross-sectional analysis

**DOI:** 10.3389/fpubh.2026.1819901

**Published:** 2026-06-18

**Authors:** Nesrullah Ayşin, Fatma Güdücü Tüfekci, Abdullah Adıyaman, Veysel Can, Mehmet Bulduk

**Affiliations:** 1 Hakkari University, Hakkari, Türkiye; 2 Ataturk Universitesi, Erzurum, Türkiye; 3 Department of Pediatric Nursing, Institute of Health Sciences, Atatürk University, Erzurum, Türkiye; 4 Department of Nursing, Faculty of Health Sciences, Erzurum Technical University, Erzurum, Türkiye; 5 Van Yuzuncu Yil Universitesi, Van, Türkiye

**Keywords:** disaster preparedness, hearing impairment, protective factors, psychological resilience, public health, vulnerable families

## Abstract

**Background:**

Families of children with disabilities are considered vulnerable populations in disaster contexts due to communication barriers, increased caregiving demands, and limited access to emergency resources. Psychological resilience, defined as the capacity to adapt positively in the face of adversity, may function as a protective factor influencing disaster preparedness behaviors. However, evidence regarding the role of parental psychological resilience in shaping disaster preparedness among parents of children with hearing impairment remains limited.

**Methods:**

This cross-sectional descriptive study was conducted between December 2023 and January 2025 with 183 parents of children with hearing impairment. Data were collected using a Parent–Child Information Form, the Adult Psychological Resilience Scale, and the Disaster Preparedness Scale. Descriptive statistics, independent samples *t*-test, one-way ANOVA, Pearson correlation analysis, and hierarchical multiple regression analysis were performed using SPSS 29.0. Statistical significance was set at *p* < 0.05.

**Results:**

Psychological resilience was positively and significantly associated with disaster preparedness (*r* = 0.227, *p* = 0.002). Regression analysis indicated that psychological resilience was significantly associated with disaster preparedness levels (*β* = 0.23, *B* = 0.16, *p* = 0.002). Parents with higher resilience scores demonstrated higher preparedness levels. Education level, prior disaster training, and home preparedness behaviors were also significantly associated with both resilience and preparedness. The effect size of resilience was low, suggesting that disaster preparedness is a multidimensional construct influenced by both psychological and socio-structural factors.

**Conclusion:**

Psychological resilience is a significant but not solely sufficient protective factor for disaster preparedness among parents of children with hearing impairment. Integrating psychosocial strengthening components into disaster preparedness interventions may enhance behavioral readiness in vulnerable families. Public health strategies should prioritize inclusive, family-centered, and accessible preparedness programs to support resilience and reduce disaster-related vulnerabilities.

## 1 Introduction

Disasters are events that combine the destructive power of nature and cause serious disruptions to social order (1). Various types of disasters, such as earthquakes, floods, storms, and forest fires, can radically change the lives of individuals and communities, leading to serious psychological, social, and economic consequences (2–4). Türkiye is among the countries at high risk for natural disasters, particularly earthquakes, due to its geographical location (5). The February 6, 2023 Kahramanmaraş earthquakes, which affected millions of people, caused widespread physical, social, and psychological destruction and brought disaster preparedness back to the forefront of public health priorities (6). Such large-scale disasters affect not only the physical safety of individuals but also the functioning of family systems, caregiving processes, and psychosocial wellbeing (7). Families with children with disabilities are particularly vulnerable in disaster contexts, as they face multiple challenges including communication barriers, increased caregiving burden, limited access to accessible services, and disruption of support systems (8). In families with children with hearing impairments, additional challenges arise in accessing early warning systems, maintaining communication during emergencies, and managing evacuation processes (9). These conditions increase parental responsibility for ensuring child safety while also complicating disaster preparedness processes (10). While individuals’ responses to such events vary, it has been reported that persons with disabilities are among the most vulnerable groups and are more severely affected during disasters than those without disabilities (11, 12). This disproportionate impact can be explained within the framework of social vulnerability theory, which emphasizes that disaster risk is shaped not only by hazard exposure but also by pre-existing social, economic, and functional inequalities (13, 14). Families of children with disabilities often face layered vulnerabilities, including increased caregiving demands, dependence on assistive resources, and limited access to inclusive emergency services (15). Empirical evidence indicates that these families encounter greater challenges in evacuation, reduced access to timely and accessible information, and higher risk of adverse outcomes during disasters (7, 8, 16). Studies indicate that the likelihood of death during a disaster is two to four times higher for persons with disabilities (17). Disaster preparedness is defined as the capacity to take precautions against potential hazards in advance, develop evacuation plans, and demonstrate appropriate behavior during a disaster (18). Despite the importance of being prepared for disasters, people with disabilities do not feel confident about preparing (19) and are less likely to participate in preparedness actions due to the interaction of various individual and social factors (20, 21). Consequently, they have fewer options and opportunities to access and use risk information and preparedness resources (18). Hearing-impaired individuals, in particular, may face significant barriers in accessing disaster warning systems, communicating during evacuation processes, and benefiting from emergency services (22). This situation directly affects the disaster preparedness levels of parents with hearing-impaired children, and they experience confusion because they are dependent on their families (23). It has been stated that parents need information, education, and training to meet their children’s special needs and ensure their safety (8). Having a child with hearing impairment already brings certain challenges in daily life; it requires parents to make constant efforts in areas such as communication barriers, educational processes, and social integration (24). This particular situation has the potential to profoundly affect parents’ overall psychological wellbeing and coping strategies (25). Psychological resilience, defined as individuals’ ability to demonstrate positive adaptation skills and overcome difficulties in the face of adverse life events, trauma, or stress (26), is a critical factor that directly affects individuals’ ability to adapt before, during, and after a disaster, cope with traumatic experiences, and return to their normal lives (27, 28). Individuals with high levels of psychological resilience are observed to experience lower levels of psychopathological symptoms such as depression, anxiety, and post-traumatic stress disorder after a disaster (29). Parents’ psychological resilience has a significant impact on their children’s overall wellbeing and adaptation processes (28). This is even more pronounced for parents of children with hearing impairments, as these parents have to cope with additional stress factors related to their children’s special educational needs, health issues, and social integration processes (30). In this context, parents’ levels of psychological resilience may affect their capacity to develop, implement, and maintain personal and family preparedness plans for disasters (28, 31). Parents with high psychological resilience may be more likely to engage in proactive behaviors such as receiving disaster training, creating evacuation plans, and preparing emergency kits (32). Children with hearing impairments, in particular, may have difficulty perceiving auditory warning systems (sirens, alarm sounds, etc.), which may increase parents’ concerns about their children’s safety (33). At this point, the potential impact of the psychological resilience levels of parents with hearing-impaired children on their disaster preparedness is an important issue that needs to be examined. While a growing body of research has examined disaster preparedness among persons with disabilities, studies focusing specifically on families with children with disabilities remain relatively limited. Existing evidence suggests that parents of children with disabilities often experience increased caregiving demands, heightened safety concerns, and greater challenges in accessing disaster-related information and resources (8, 15). These families may require individualized preparedness strategies that address both the child’s functional needs and the family’s capacity to respond effectively during emergencies. Moreover, parental preparedness is influenced not only by knowledge and resources but also by psychological and emotional factors, including stress, perceived vulnerability, and coping capacity (31). Despite these insights, there is still a lack of focused research examining how parents’ psychological characteristics, particularly resilience, shape preparedness behaviors in the context of childhood disability. However, the existing literature on this subject, particularly in the Turkish context, lacks studies that comprehensively address the relationship between the psychological resilience of parents of children with hearing impairments and their disaster preparedness. This study aims to fill this gap in the literature, providing a deeper understanding of the disaster preparedness processes of families with children with special needs and guiding future research in this area. Ultimately, this research is expected to provide valuable information not only for the academic literature but also for policymakers and civil society organizations to develop evidence-based interventions that are applicable in the field.

## 2 Method

### 2.1 Type of study

This is a cross-sectional descriptive study. The study was registered on ClinicalTrials.gov (NCT06542081) prior to data collection. The dates reported in the registry reflect the initially planned study timeline; however, the actual data collection was conducted between December 2024 and January 2025.

### 2.2 Location and time of the study

The study was conducted between September 2024 and January 2025 at a center affiliated with the Provincial Directorate of National Education in a province in eastern Turkey that provides education for children with hearing and speech impairments.

### 2.3 Study population and sample

The parents of children with hearing impairments at the special rehabilitation center where the data was collected constituted the population of the study. Participants were recruited from among parents of children with hearing impairments receiving care at rehabilitation centers in a single province in eastern Turkey using a non-probability convenience sampling approach. Based on the information obtained, it was determined that there were 214 children with hearing impairments, and that a sample size of 137 parents would be sufficient for a known population with a 95% confidence level and a 5% margin of error. In order to increase the power of the study, an attempt was made to reach the entire sample, but 196 parents were interviewed. Six of the interviewed parents were excluded because they did not want to participate, and seven were excluded because they did not complete the questionnaire forms. The research process was completed with 183 parents. Parents in the study had children across a broad age range (0–18 years), including early childhood, middle childhood, and adolescence. However, age-specific subgroup analyses were not conducted, which should be considered when interpreting the findings.

### 2.4 Inclusion and exclusion criteria for the study

#### 2.4.1 Inclusion criteria

The inclusion criteria were as follows:

- Being a parent of a child receiving education at a school for the hearing impaired
- Being able to read and understand written Turkish sufficiently to complete the questionnaire independently
- Being willing to participate in the study

#### 2.4.2 Exclusion criteria

Participants were excluded if they:

- Were unable to complete the questionnaire independently due to cognitive, language, or communication difficulties
- Provided incomplete or inconsistent responses
- Declined to participate or withdrew during data collection

### 2.5 Data collection tools

The Parent–Child Descriptive Information Form, consisting of 25 questions and prepared using the literature to determine socio-demographic characteristics, the Adult Psychological Resilience Scale (APRS), consisting of 21 questions, and the Disaster Preparedness Scale, consisting of 13 questions, were used as other data collection tools.

#### 2.5.1 Parent–child descriptive information form

Drawing on the literature, a 25-question form was created to determine socio-demographic characteristics such as age, gender, income status, chronic disease status, number of children, and where the family lives (18, 25, 28, 30).

#### 2.5.2 Adult psychological resilience scale (APRS)

The scale consists of 21 items in total. The five-point Likert scale is rated between “Describes me completely (5)” and “Does not describe me at all (1)”. Higher scores indicate higher psychological resilience. The Turkish validity and reliability study of the scale was conducted by Arslan (34) with 470 participants. In the present study, the scale was applied to parents aged 18 years and older. The internal consistency coefficient of the scale was found to be 0.94, and the test–retest coefficient was 0.85. As a result of the validity and reliability studies, it was found that the scale can be used to assess psychological resilience in adults in our country. In the study, the Cronbach Alpha internal consistency coefficient was determined to be 0.81.

#### 2.5.3 Disaster preparedness scale (DPS)

The scale developed by Şentuna and Çakı (2020) consists of 13 questions and four subscales. The scale is rated as 1 = Definitely no, 2 = No, 3 = Yes, and 4 = Definitely yes. It has been stated that as the score obtained from the scale increases, the level of disaster preparedness rises. The four factors are named as follows: Disaster Physical Protection (DPP), Disaster Planning (DP), Disaster Assistance (DA), Disaster Warning and Signals (DWS). The Cronbach’s Alpha internal consistency coefficient for the scale was reported to be 0.82. (35). In the study, the Cronbach’s Alpha internal consistency coefficient was determined to be 0.83.

### 2.6 Data collection

The data for the study were collected by the researcher using data collection tools that took 15–20 min to complete, administered to parents in the guidance room of the rehabilitation center. The questionnaires were administered to participants face-to-face on a voluntary basis. Before the application, participants were informed about the purpose, scope, and confidentiality principles of the study, and their verbal and written consent was obtained. The questionnaire form was prepared to include understandable and non-leading questions, and appropriate conditions were provided for participants to give accurate and sincere answers.

### 2.7 Data analysis

The data obtained in the study were analyzed using the SPSS 29.0 software package. Descriptive statistics were primarily used to evaluate the data; counts, percentages, medians, means, and standard deviations were calculated. The normality of scale scores was examined using kurtosis and skewness values; since they fell within the ±2.5 range, it was accepted that the data met the assumption of normal distribution. To determine the difference between groups, the *t*-test for independent groups and one-way analysis of variance (One Way ANOVA) were used. Post-hoc tests were used to determine which groups contributed to the differences found to be significant in the ANOVA results. Pearson correlation analysis was applied to examine the relationship between two variables. A statistical significance level of *p* < 0.05 was accepted for the results obtained. For effect size, eta-squared (*η*^2^) values were interpreted as 0.01 = small, 0.06 = medium, 0.14 = large effect; Cohen’s *d* values were interpreted as 0.20 = small, 0.50 = medium, 0.80 = large effect. Effect sizes for correlation coefficients were interpreted based on Cohen’s criteria, where *r* = 0.10 indicates a small effect, *r* = 0.30 a medium effect, and *r* = 0.50 a large effect (36, 37). A hierarchical multiple linear regression analysis was conducted in two blocks to examine factors associated with DPS total score. In the first block, sociodemographic variables (age, education, employment status, family type, income status, consanguineous marriage, and chronic illness) were included as control variables. In the second block, disaster-related variables (sign language training, prior disaster experience, household disaster preparedness, disaster education) and APRS total score were added to the model. This approach allowed the evaluation of the additional variance explained by the second block after controlling for sociodemographic factors. Because the Durbin–Watson statistic fell below the ideal range, the final regression model was reanalyzed using HC3 robust standard errors, and the results were reported separately as a sensitivity analysis.

### 2.8 Ethical principles

As this research was conducted on human participants, it was carried out in accordance with ethical principles. Before starting the research, ethical approval (Decision no: 2023/08–08, Date: 11.08.2023) was obtained from the Non-Interventional Ethics Committee of Van Yüzüncü Yıl University, and the necessary written permissions for collecting the research data were obtained from the relevant institution. Parents were informed about the purpose, scope, and confidentiality principles of the research, and informed consent was obtained on a voluntary basis. The participants’ identity information was kept confidential, and the data obtained was used solely for scientific purposes. The security of the data was ensured, and it was not shared with third parties. Throughout the research process, the ethical principles outlined in the Declaration of Helsinki and the provisions of the “Personal Data Protection Law (PDPL)” were adhered to.

## 3 Results

Descriptive analyses conducted to determine sociodemographic characteristics revealed that 79.8% of the parents participating in the study were mothers, 35% were aged 24–31 and 35% were aged 32–39, 31.1% were middle school graduates, 76% were not working, 80.3% had a nuclear family, 89.6% lived in an urban area, 51.4% had an income equal to their expenses, 45.4% had two children, and 59% had no kinship with their spouse. Of the parents, 74.9% had no chronic illness, 69.4% had no other disabled family member, 48.6% were shocked by their child’s hearing impairment diagnosis, 76.5% felt their lives were affected by having a child with hearing impairment, 68.3% had no problems in their relationship with their spouse, 63.9% had not received sign language training, and 92.9% engaged in daily activities with their child with hearing impairment. Regarding the parents, 71% had previously been exposed to a disaster, 57.4% knew how to protect themselves and their children during disasters, 65.6% did not have a disaster preparedness plan at home, and 71% had not received any disaster-related training. When the descriptive characteristics of the children were examined, it was determined that 42.6% were between the ages of 10–18, 51.9% were female, 76.5% had no other chronic illness, and 63.4% of the children had a congenital hearing impairment (Table 1).

**Table 1:** Socio-demographic characteristics of parents and children (*N* = 183).

Variables		*N*	%
Parent			
	Mother	146	79.8
	Father	37	20.2
Age			
	18–24	19	10.4
	25–31	64	35.0
	32–39	64	35.0
	40–48	36	19.6
Educational status			
	Elementary school	61	33.3
	Middle school	57	31.2
	High school	34	18.6
	University	31	16.9
Employment status			
	Employed	44	24.0
	Not working	139	76.0
Family type			
	Nuclear	147	80.3
	Large	36	19.7
Living area			
	Urban	164	89.6
	Rural	19	10.4
Income status			
	Income is less than expenses	65	35.5
	Income equals expenses	94	51.4
	Income exceeds expenses	24	13.1
Number of children			
	1	32	17.5
	2	83	45.4
	3 and above	68	37.2
Relationship with spouse			
	Yes	75	41.0
	No	108	59.0
Chronic disease status			
	Yes	46	25.1
	None	137	74.9
Presence of another disabled person in the family			
	Yes	56	30.6
	No	127	69.4
Family reactions after diagnosis			
	I did not want to accept it	27	14.8
	I was shocked	89	48.6
	I blamed myself or my spouse	42	23.0
	I blamed the doctor	18	9.8
	I accepted it	7	3.8
Effects of hearing loss on family life			
	Yes	140	76.5
	No	43	23.5
Relationship with spouse after hearing impairment (psychological, social, etc.)			
	Yes	58	31.7
	No	125	68.3
Sign language training status			
	Yes	66	36.1
	No	117	63.9
Parent–child activity during the day			
	Yes	170	92.9
	No	13	7.1
Previous disaster experience			
	Yes	130	71.0
	No	33	29.0
Knowledge of protecting oneself and one’s child in disasters			
	Yes	105	57.4
	No	78	42.6
Disaster preparedness at home			
	Yes	63	34.4
	No	120	65.6
Status of receiving disaster-related training			
	Yes	53	29.0
	No	130	71.0
Disaster education resource (*n* = 53)			
	School/course	31	58.5
	TV	14	26.4
	Social media	8	15.1
Child’s age			
	0–4 years	35	19.1
	5–9 years	70	38.3
	10–18 years	78	42.6
The child’s gender			
	Girl	95	51.9
	Male	88	48.1
Additional chronic illness in the child			
	Yes	43	23.5
	No	140	76.5
The onset of hearing loss			
	Congenital	116	63.4
	Later	67	36.6

*N*, Number of participants; %, Percentage.

The descriptive characteristics of the scales are presented in Table 2. The analysis revealed that the skewness and kurtosis values were within normal limits (± 2.5). In the Pearson correlation analysis between the scales, a statistically significant relationship was found between the total scores of APRS and DPS (*r* = 0.227, *p* = 0.002). Furthermore, a statistically significant relationship was found between the APRS total score and the DPP (*r* = 0.247, *p* < 0.001) and DA (*r* = 0.183, *p* = 0.013) subscales, respectively (Table 2).

**Table 2:** Correlation matrix and descriptive statistics for the scales

**Scales**	**X ± SD** **Median (Min/Max)**	**APRS** 75.01 (9.84) 76 (39-96)	
		**r**	**p**
**DPS**	29.18 **±** 9.84 29 (13-44)	.227*	**0.002**
**DPP**	10.81 **±** 2.95 11 (5-17)	.247**	**<.001**
**DP**	6.40 **±** 2.56 6 (3-11)	0.112	0.133
**DA**	8.14 **±** 1.71 8 (3-11)	.183*	**0.013**
**DWS**	3.82 ±2.03 3 (2-8)	.125	.092
	**Skewness**	**Kurtosis**	
**APRS**	-0.561	0.699	
**DPS**	0.027	-0.867	
**DPP**	-0.149	-0.849	
**DP**	-0.183	-1.608	
**DA**	-0.452	0.38	
**DWS**	0.499	-1.368	

DPS: Disaster Preparedness Scale, DPP: Disaster Physical Protection, DP: Disaster Planning, DA: Disaster Assistance, DWS: Disaster Warning and Signals, APRS: Adult Psychological Resilience Scale, X̄: Mean, SD: Standard deviation, r: Pearson correlation test, *: p<.05, **: p<.001.

A two-block hierarchical multiple linear regression analysis was conducted to examine the variables associated with the total DPS score. In the first block, sociodemographic variables were included in the model. Model 1 was found to be statistically significant (*F*(12,170) = 2.102, *p* = 0.019). Model 1 explains 12.9% of the variance in the total DPS score. In Model 2, disaster-related variables and the total APRS score were added to the model. The explanatory power of the model increased to a statistically significant level (Δ*R*^2^ = 0.108, Δ*F*(4,166) = 5.848, *p* < 0.001). Model 2 explains 23.7% of the variance in the total DPS score (*F*(16,166) = 3.219, *p* < 0.001, adjusted *R*^2^ = 0.163). The Durbin–Watson value was determined to be 1.209 (Table 3).

**Table 3:** Hierarchical multiple linear regression analysis examining factors associated with DPS total score.

Independent variables		*B*	95% CI lower	95% CI upper	SE	*β*	*t*	*p*	*r* ^1^	*r* ^2^	VIF
Model 1 Sociodemographic variables											
Constant		28.388	23.937	32.839	2.255	–	12.590	**<0.001**	–	–	–
Age	25–31 years	1.914	−1.761	5.589	1.862	0.131	1.028	0.305	0.136	0.079	3.161
	32–39 years	1.865	−1.691	5.421	1.801	0.127	1.035	0.302	0.066	0.079	2.959
	≥40 years	−1.324	−5.433	2.786	2.082	−0.075	−0.636	0.526	−0.179	−0.049	2.746
Education	Secondary school	1.771	−1.150	4.692	1.480	0.118	1.197	0.233	0.111	0.091	1.883
	High school	0.828	−2.729	4.386	1.802	0.046	0.460	0.646	0.054	0.035	1.970
	University	1.815	−1.825	5.455	1.844	0.098	0.984	0.326	0.105	0.075	1.919
Working status		1.783	−0.827	4.393	1.322	0.109	1.348	0.179	0.157	0.103	1.280
Family type		−2.875	−5.479	−0.272	1.319	−0.164	−2.180	**0.031**	−0.151	−0.165	1.102
Income status	Income equal to expenses	−0.985	−3.266	1.297	1.156	−0.071	−0.852	0.395	−0.040	−0.065	1.338
	Income greater than expenses	0.195	−3.172	3.562	1.706	0.009	0.114	0.909	0.057	0.009	1.329
Consanguineous marriage status		−1.619	−4.024	0.785	1.218	−0.114	−1.329	0.186	−0.162	−0.101	1.439
Chronic disease in parent/spouse		0.017	−2.478	2.513	1.264	0.001	0.014	0.989	−0.014	0.001	1.206
Model 2 Sociodemographic, disaster-related variables and APRS											
Constant		13.285	3.534	23.035	4.938	–	2.690	**0.008**	–	–	–
Age	25–31 years	2.772	−0.863	6.407	1.841	0.189	1.506	0.134	0.136	0.116	3.444
	32–39 years	4.146	0.487	7.805	1.853	0.283	2.237	**0.027**	0.066	0.171	3.490
	≥40 years	1.046	−3.146	5.239	2.123	0.060	0.493	0.623	−0.179	0.038	3.183
Education	Secondary school	0.691	−2.179	3.561	1.454	0.046	0.476	0.635	0.111	0.037	2.024
	High school	0.019	−3.392	3.431	1.728	0.001	0.011	0.991	0.054	0.001	2.018
	University	−0.765	−4.549	3.019	1.916	−0.041	−0.399	0.690	0.105	−0.031	2.309
Working status		1.286	−1.245	3.818	1.282	0.079	1.003	0.317	0.157	0.078	1.341
Family type		−2.693	−5.210	−0.176	1.275	−0.153	−2.112	**0.036**	−0.151	−0.162	1.148
Income status	Income equal to expenses	−0.678	−2.893	1.537	1.122	−0.049	−0.604	0.546	−0.040	−0.047	1.405
	Income greater than expenses	0.762	−2.446	3.971	1.625	0.037	0.469	0.640	0.057	0.036	1.344
Consanguineous marriage status		−1.654	−3.949	0.642	1.163	−0.117	−1.422	0.157	−0.162	−0.110	1.461
Chronic disease in parent/spouse		0.486	−1.921	2.894	1.219	0.030	0.399	0.691	−0.014	0.031	1.250
All family members received sign language training		−0.897	−3.008	1.215	1.069	−0.062	−0.838	0.403	−0.111	−0.065	1.178
Previous disaster experience		2.500	0.294	4.706	1.117	0.163	2.238	**0.027**	0.212	0.171	1.147
Disaster-related education		3.121	0.788	5.454	1.182	0.203	2.641	**0.009**	0.258	0.201	1.283
APRS		0.158	0.041	0.275	0.059	0.222	2.674	**0.008**	0.227	0.203	1.504

Model 1: *F*(12,170) = 2.102, *p* = 0.019, *R*^2^ = 0.129, adjusted *R*^2^ = 0.068. Model 2: *F*(16,166) = 3.219, *p* < 0.001, *R*^2^ = 0.237, adjusted *R*^2^ = 0.163. The addition of disaster-related variables and APRS total score in Model 2 significantly improved the explained variance, Δ*R*^2^ = 0.108, Δ*F*(4,166) = 5.848, *p* < 0.001. Durbin–Watson = 1.209.

B, unstandardized regression coefficient; SE, standard error; *β*, standardized regression coefficient; CI, confidence interval; *r*^1^, zero-order correlation; *r*^2^, partial correlation; VIF, variance inflation factor. The dependent variable was DPS total score. Reference categories were 18–24 years for age, primary school for education, income lower than expenses for income status, and the reference categories of binary variables were determined according to the coding scheme. Higher APRS total scores were significantly associated with higher DPS total scores after controlling for sociodemographic and disaster-related variables.

Since the Durbin–Watson value fell below the ideal range, the final model was reanalyzed using HC3 robust standard errors. This sensitivity analysis was conducted for Model 2, the final regression model (Model 2: *F*(16,166) = 3.219, *p* < 0.001, *R*^2^ = 0.237, adjusted *R*^2^ = 0.163). The Durbin–Watson statistic for Model 2 was found to be 1.20876. The robust analysis showed a significant positive association between the total APRS score and the total DPS score (*B* = 0.158, robust SE = 0.070, *p* = 0.026). Similarly, the previous findings regarding disaster experience (*B* = 2.500, robust SE = 1.181, *p* = 0.036) and disaster-related training (*B* = 3.121, robust SE = 1.403, *p* = 0.027) also retained their significance. These results support the relationship between psychological resilience and disaster preparedness (Appendix 1).

In Model 2, the variables statistically significantly associated with the total DPS score were: being in the 32–39 age group, family type, having previously experienced a disaster, having received disaster-related education, and the total APRS score. Accordingly, it was determined that the total DPS scores of individuals in the 32–39 age group were higher than those of the reference age group (*B* = 4.146, *β* = 0.283, *p* = 0.027). The family type variable showed a significant relationship with the total DPS score. It was determined that individuals with a nuclear family structure had higher total DPS scores compared to those in extended families (*B* = −2.693, *β* = −0.153, *p* = 0.036). Individuals who had previously experienced a disaster had higher total DPS scores (*B* = 2.500, *β* = 0.163, *p* = 0.027). Individuals who received disaster-related education also had higher total DPS scores (*B* = 3.121, *β* = 0.203, *p* = 0.009). The APRS total score, however, was positively and significantly associated with the DPS total score (*B* = 0.158, *β* = 0.222, *p* = 0.008). This finding indicates that as psychological resilience increases, so does the level of disaster preparedness (Table 3).

Table 4 compares APRS and DPS scores according to sociodemographic variables. Statistically significant differences were found in APRS scores for the variables of age, educational status, presence of chronic illness, receipt of sign language training, and receipt of disaster-related training. Regarding DPS scores, significant differences were identified in the variables of age, educational status, employment status, family type, marital status, disaster experience, household disaster preparedness, and disaster-related training. In contrast, no significant differences were found in variables such as income level, number of children, place of residence, and daily parent–child interaction. The effect sizes obtained were mostly small to moderate (Table 4).

**Table 4:** Comparison of APRS and DPS scores according to socio-demographic characteristics of parents and corresponding statistical test results.

Variables	*N*	APRS		DPS	
		Mean ± SD	Statistical test (*t*/*F*) (df) *p* value d/η²	Mean ± SD	Statistical test (*t*/*F*) (df) *p* value d/η²
Parent					
Mother	146	75.49 **±** 9.09	*t*_(181)_ = 1.10 *p* = 0.276 *d* = 0.24	28.95 **±** 6.83)	*t*_(181)_ = 0.89 *p* = 0.371 *d* = −0.16
Father	37	73.11 (12.33)		30.11 ± 7.66)	
Age					
18–24	19	79.32 **±** 8.83^a^	*F*_(3,179)_ = 8.71 *p* < 0.001^**^ η^2^ = 0.127	27.47 ± 8.26^a, b^	*F*_(3,179)_ = 8.71 *p* = 0.035^*^ η^2^ = 0.047
25–31	64	78.63 **±** 7.88^a^		30.48 **±** 6.95^a^	
32–39	64	72.55 **±** 10.69^b^		29.81 **±** 7.47^a, b^	
40–48	36	70.69 **±** 9.07^b^		26.67 **±** 4.4^b^	
Educational status					
Elementary	61	70.16 **±** 8.20^a^	*F*_(3.179)_ = 8.71 *p* < 0.001^**^ η^2^ = 0.198	26.85 **±** 6.16^a^	*F*_(3.62)_ = 8.71 *p* < 0.014^*^ η^2^ = 0.057
Middle school	57	76.44 **±** 9.03^b^		30.33 **±** 7.39^b^	
High School	34	74.15 **±** 11.10^a, b^		29.97 **±** 7.68^b^	
University	31	82.87 **±** 7.03^c^		30.81 **±** 6.12^b^	
Employment status					
Employed	44	74.61 **±** 10.92	*t*_(181)_ = −0.31 *p* = 0.760 *d* = −0.05	31.14 **±** 7.33	*t*_(181)_ = 2.14 *p* = 0.033^*^ d = 0.37
Not working	139	75.14 **±** 9.52		28.57 **±** 6.80	
Family type					
Nuclear	147	74.49 **±** 9.66	*t*_(181)_ = −1.45 *p* = 0.148 *d* = −0.27	29.71 **±** 7.04	*t*_(181)_ = 2.06 *p* = 0.041^*^ d = 0.38
Large	36	77.14 **±** 10.45		27.06 **±** 6.47	
Place of residence					
Urban	164	75.16 **±** 9.76	*t*_(181)_ = 0.60 *p* = 0.553 *d* = 0.14	29.19 **±** 7.08	*t*_(181)_ = 0.02 *p* = 0.985 *d* = 0.00
Rural	19	73.74 **±** 10.71		29.16 **±** 6.41	
Income level					
Income is less than expenses	65	75.**03 ±** 8.15	*F*_(2.180)_ = 0.03 *p* = 0.968 *η*^2^ = 0.000	29.20 **±** 7.61	*F*_(2.180)_ = 0.32 *p* = 0.723 *η*^2^ = 0.004
Revenue equals expenses	94	75.11 **±** 10.28		28.91 **±** 6.67	
Revenue exceeds expenses	24	74.54 **±** 12.38		30.21 **±** 6.71	
Number of children					
1	32	77.19 **±** 11.89	*F*_(2.180)_ = 2.08 *p* = 0.129 *η*^2^ = 0.022	29.09 **±** 5.95	*F*_(2.180)_ = 2.25 *p* = 0.109 *η*^2^ = 0.024
2	83	75.63 **±** 10.34		30.29 **±** 6.62	
3 and above	68	73.24 **±** 7.81		27.88 **±** 7.72	
Spousal relationship status					
Yes	75	73.97 **±** 10.14	*t*_(181)_ = −1.19 *p* = 0.236 *d* = −0.18	27.83 **±** 7.15	*t*_(181)_ = −2.21 ***p* = 0.028** *d* = −0.33
No	108	75.73 **±** 9.61		30.13 **±** 6.76	
Chronic disease status					
Yes	46	72.43 **±** 10.11	*t*_(181)_ = −2.07 *p* = 0.040^*^ d = −0.35	29.02 **±** 7.37	*t*_(181)_ = −0.18 *p* = 0.855 *d* = −0.03
None	137	75.88 **±** 9.63		29.24 **±** 6.89	
Presence of another disabled individual in the family					
Yes	56	76.07 **±** 8.96	*t*_(181)_ = 0.97 *p* = 0.335 *d* = 0.16	28.11 ± 6.87	*t*_(181)_ = −1.39 *p* = 0.167 *d* = −0.22
None	127	74.54 **±** 10.21		26.89 **±** 6.03	
Family reactions after diagnosis					
I did not want to accept it	27	74.78 **±** 9.68	*F*_(4.178)_ = 0.09 *p* = 0.986 *η*^2^ = 0.002	31.33 **±** 5.50	*F*_(4.178)_ = 1.70 *p* = 0.153 *η*^2^ = 0.037
I was shocked	89	75.17 **±** 9.52		28.85 **±** 6.83	
I blamed myself/my spouse	42	74.55 **±** 10.73		29.71 **±** 7.43	
I blamed the doctor	18	76.00 ± 10.58		26.06 **±** 8.98	
I accepted it	7	74.14 **±** 9.79		30.00 **±** 3.00	
Effects of hearing loss on family life					
Yes	140	74.79 **±** 10.37	*t*_(181)_ = 1.65 *p* = 0.578 *d* = −0.10	29.66 **±** 7.00	*t*_(181)_ = 1.65 *p* = 0.100 *d* = 0.29
No	43	75.74 **±** 7.96		27.65 **±** 6.83	
Relationship after hearing impairment (psychological, social, etc.)					
Yes	58	73.02 **±** 11.95	*t*_(181)_ = −1.88 *p* = 0.062 *d* = −0.30	29.34 **±** 7.20	*t*_(181)_ = 0.21 *p* = 0.835 *d* = 0.03
No	125	75.94 **±** 8.60		29.11 **±** 6.92	
Sign language training status					
Yes	66	77.33 **±** 8.70	*t*_(181)_ = 2.43 *p* = 0.016^*^ d = 0.37	28.15 **±** 6.39	*t*_(181)_ = −1.51 *p* = 0.134 *d* = −0.23
No	117	73.70 **±** 10.24		29.77 **±** 7.28	
Parent–child activity during the day					
Yes	170	74.94 **±** 9.84	*t*_(181)_ = −0.35 *p* = 0.730 *d* = −0.10	29.12 **±** 6.98	*t*_(181)_ = −0.48 *p* = 0.635 *d* = −0.14
No	13	75.92 **±** 10.23		30.08 **±** 7.48	
Disaster experience					
Yes	130	75.18 **±** 9.75	*t*_(181)_ = 0.36 *p* = 0.722 *d* = 0.06	30.13 **±** 7.00	*t*_(181)_ = 2.92 *p* = 0.004^*^ d = 0.48
No	53	74.60 **±** 10.16		26.87 **±** 6.48	
Knowledge of protecting oneself and one’s child in disasters					
Yes	105	76.09 **±** 9.81	*t*_(181)_ = 1.72 *p* = 0.087 *d* = 0.26	29.32 **±** 7.22	*t*_(181)_ = 0.31 *p* = 0.755 *d* = 0.05
No	78	73.56 **±** 9.76		29.00 **±** 6.73	
Home disaster preparedness status					
Yes	63	76.35 **±** 10.05	*t*_(181)_ = 1.34 *p* = 0.183 *d* = 0.21	33.56 **±** 6.66	*t*_(181)_ = 6.85 *p* < 0.001^**^ d = 1.07
No	120	74.31 **±** 9.70		26.89 **±** 6.03	
Status of receiving disaster-related training					
Yes	53	78.74 **±** 7.04	*t*_(181)_ = 3.36 *p* < 0.001^**^ d = 0.55	32.00 **±** 7.50	*t*_(181)_ = 3.59 *p* < 0.001^**^ d = 0.58
No	130	73.49 **±** 10.43		28.04 **±** 6.47	

Number of participants; DPS, Disaster Preparedness Scale; APRS, Adult Psychological Resilience Scale; X̄, Mean; SD, Standard deviation; *t*, Independent samples *t*-test; *F*, One-way ANOVA; df, Degrees of freedom. Cohen’s *d* values are reported for independent samples *t*-tests, and eta-squared (*η*^2^) values are reported for one-way ANOVA. Effect sizes were interpreted as small (*d* = 0.20, *η*^2^ = 0.01), medium (*d* = 0.50, *η*^2^ = 0.06), and large (*d* = 0.80, *η*^2^ = 0.14). ^*^p < 0.05, ^**^p < 0.001.

## 4 Discussion

This study examined the effect of psychological resilience levels of parents of hearing-impaired children on their disaster preparedness status and determined that psychological resilience is associated with disaster preparedness. Beyond being an individual trait, psychological resilience can also be understood as a dynamic and context-dependent process that evolves through interaction with environmental demands and caregiving responsibilities (38, 39). In the context of disaster preparedness, parents’ resilience is not only reflected in their internal coping capacity but also in how they actively organize, adapt, and implement protective strategies for their children. Especially in families of children with hearing impairments, resilience may manifest through ongoing efforts to anticipate risks, ensure effective communication, and maintain safety under conditions of uncertainty (8). This process-oriented perspective highlights that resilience is continuously shaped by caregiving experiences and situational challenges rather than remaining a fixed personal characteristic. The findings are consistent with the current literature, which shows that the capacity to adapt and cope with disasters is closely related not only to logistical preparedness or knowledge but also to the psychological resources possessed by the individual (38, 40, 41). Current syntheses on the psychological determinants of disaster preparedness emphasize that preparedness is related to self-regulation, self-efficacy, stress tolerance, and proactive coping processes, thereby reinforcing this alignment (32, 42). Therefore, the main contribution of this study is to reveal psychological resilience in parents of children with hearing impairments not as a “secondary” variable, but as one of the multi-level capacity domains that shape preparedness.

This multi-level interpretation becomes more meaningful when considered within the protective factors framework. The protective factors approach emphasizes personal and environmental resources that enable individuals to maintain their functioning in the face of risky life events (43). According to this model, psychological resilience supports the individual’s perception of threats as manageable by incorporating characteristics such as hope, self-efficacy, problem-solving skills, and cognitive flexibility (39). Disaster preparedness behaviors are closely related to self-regulation processes that enable the perception of “threat” to be transformed into an “action plan,” as an outcome of this cognitive assessment process (32, 39). Therefore, in the current study, the increase in preparedness as resilience increases suggests that parents can activate their proactive coping repertoires even under conditions of uncertainty and transform preparedness from an avoidance area into action.

This line of explanation also aligns with Lazarus and Folkman’s Stress and Coping Theory, which clarifies how psychological processes translate into behavior. According to this model, when individuals encounter a potential threat, they first assess the situation and then review their coping resources (44). Psychologically self-sufficient individuals tend to adopt problem-focused strategies rather than avoidance (45). Since disaster preparedness involves direct problem-focused behaviors such as planning, gathering information, and obtaining protective equipment, it is theoretically expected that these behaviors would be more common among resilient parents (18, 45). Therefore, it can be considered that the resilience–preparedness link triggers preparedness behaviors by limiting the transformation of threat perception into paralyzing anxiety in the primary assessment and strengthening the belief that “I can cope” in the secondary assessment. However, the fact that the effect of psychological resilience, while meaningful, remains low is important in that it shows that disaster preparedness is a multi-component structure that cannot be explained by a single psychological capacity (46). In line with this, the hierarchical multiple regression analysis indicated that psychological resilience remained significantly associated with disaster preparedness even after controlling for sociodemographic and disaster-related variables. However, the magnitude of this association was modest, further supporting the view that disaster preparedness is shaped by multiple interacting factors rather than a single dominant variable. Accordingly, psychological resilience should be interpreted as one of several contributing factors rather than a primary or dominant determinant of disaster preparedness. These findings should be interpreted as associations rather than causal relationships due to the cross-sectional design of the study.

The disaster literature emphasizes that, in addition to individual psychological characteristics, experience, social capital, economic opportunities, and institutional support are also determinants of preparedness (46, 47). In this context, the limited effect of resilience suggests that conditions such as accessible information channels, feasible planning opportunities, and a supportive environment are necessary for psychological capacity to translate into behavior. The modest association observed in the present study may also be partly related to the absence of child-specific clinical variables. Factors such as the severity of hearing loss, use of hearing aids or cochlear implants, and primary communication method may shape the extent of communication barriers during emergencies. These factors may function as contextual moderators that influence whether parental psychological resilience can be translated into concrete disaster preparedness behaviors. Another factor that should be considered when interpreting the findings is prior disaster experience. Although not the primary focus of the present analysis, a substantial proportion of participants reported having experienced a disaster. Previous disaster exposure may influence both preparedness behaviors and psychological resilience by shaping risk perception, learning processes, and coping strategies. Therefore, this factor may act as a contextual variable that should be taken into account when interpreting the observed associations. It should also be noted that some variables included in the analysis, such as home disaster preparedness status, may conceptually overlap with the outcome measure, as they reflect closely related aspects of disaster preparedness. Therefore, these variables should be interpreted as contextual indicators rather than independent determinants.

Indeed, relationships with sociodemographic variables also present a pattern that supports this multi-layered structure (46, 48). Findings related to the age variable are noteworthy in that younger parents demonstrate higher psychological resilience (48, 49). Another important consideration is that the majority of participants in this study were mothers. This may have influenced the findings, as caregiving roles are often more intensively assumed by mothers, particularly in families with children with disabilities (8, 50). Mothers may be more actively involved in daily caregiving, communication, and health-related decision-making processes, which could shape their perceptions of risk, preparedness behaviors, and psychological resilience differently compared to fathers. Previous studies suggest that gender differences in caregiving roles and emotional involvement may lead to variations in coping strategies and disaster preparedness behaviors (51). Therefore, the findings of this study should be interpreted with consideration of this gender distribution. Greater cognitive flexibility and openness to new information during young adulthood (52) may facilitate the internalization of disaster preparedness messages and their translation into behavior. Conversely, the increased caregiving burden and burnout that may come with age (53) can indirectly weaken preparedness by narrowing the portion of psychological resources available for disaster preparedness. Therefore, the necessity of programs integrating psychosocial support and accessible disaster education components for older age groups should be considered not merely a recommendation but a need indicated by the findings.

Another important aspect to consider when interpreting the findings is the developmental stage of the child. Children with hearing impairments at different ages require varying caregiving approaches, communication strategies, and levels of supervision (8, 50). For example, younger children tend to rely more on direct parental support and immediate physical protection, whereas older children and adolescents may require more complex communication strategies and active involvement in decision-making processes during emergencies (33, 54). These developmental differences may influence how parents perceive risks, organize preparedness behaviors, and utilize their psychological resilience in crisis situations. Therefore, parental resilience should be considered as a dynamic construct shaped by the evolving developmental needs of the child rather than a fixed individual trait.

From this perspective, the findings on educational level make the idea of preparedness shifting from “knowledge” to “capacity” more concrete. The fact that educational level is related to both psychological resilience and disaster preparedness reinforces the protective role of education. It is stated that disaster literacy is not limited to teaching risks and correct behaviors, but is also a process that develops individuals’ psychological and social resilience (55). Education can transform disaster preparedness into a sustainable set of habits by expanding the problem-solving repertoire that translates risk knowledge into behavior (32, 56). Therefore, adapted and accessible educational strategies for disadvantaged groups, such as families of children with disabilities, can serve as a common lever for both resilience and preparedness.

The “accessibility” dimension of education is becoming more visible through sign language education findings (57). The fact that parents who receive sign language education have higher psychological resilience is valuable from the perspective of the family empowerment approach (50). Increased communication capacity strengthens the parent’s sense of control, reducing uncertainty and making it easier to use coping resources more effectively (54). Family-centered care models demonstrate that active parental involvement and empowerment with knowledge enhance psychological adjustment. In this context, sign language training can be seen not only as a communicative necessity but also as a therapeutic empowerment component that prepares parents by increasing their self-efficacy and coping capacity (58). This interpretation is also consistent with calls for accessible risk communication and inclusive disaster planning in the context of disability.

The fact that working parents in the study had higher DPS scores suggests that disaster preparedness is related not only to individual psychological capacity but also to socio-economic participation and levels of social integration (59). Participation in working life can strengthen an individual’s preparedness behaviors through mechanisms such as access to information, contact with institutional communication channels, the breadth of social networks, and increased risk awareness (60). This shows that having a profession is an important factor in increasing disaster preparedness. It also highlights the need to strengthen preparedness training for non-working parents.

Similarly, the higher preparedness of parents with a nuclear family structure can be explained by more centralized and functional family decision-making processes. Disaster planning is a process that requires quick decision-making, role clarity, and clear distribution of responsibilities, and it has been suggested that these processes may become more complex in extended family structures (61). This finding shows that preparedness is shaped not only by individual intentions but also by the structural characteristics of family organization.

The significant difference in marital and kinship status can be evaluated from the perspective of social network diversity. It has been reported that families with more heterogeneous social ties may be more advantaged in terms of access to information, support, and institutional resources (62). It has been suggested that in kinship-based marriages, the social environment may be more closed and homogeneous, which may limit the flow of external information (63). In this context, the findings support the notion that disaster preparedness is a multi-layered phenomenon related not only to psychological resilience but also to social structure, family type, and social connections.

This emphasis on inclusivity makes it possible to link disaster education and disaster experience findings to the same chain of mechanisms (64, 65). The fact that parents who receive disaster education have both higher resilience and preparedness supports the mutual interaction between knowledge and self-efficacy (66, 67). Preparation training increases the sense of control, reducing anxiety caused by uncertainty and making planning behavior more likely (31). Similarly, the higher preparedness observed in parents who have previously experienced a disaster suggests that experiential learning can strengthen preparedness behavior through the concretization of risk. However, the fact that trauma can create vulnerability in some individuals while triggering growth and adaptation processes in others shows that this transformation is closely related to the presence of social support and psychological resources (68). Therefore, the effect of experience and education, when combined with psychological resilience, can be considered an integrated network of protective factors that increases the likelihood of preparedness translating into behavior.

Despite various initiatives and awareness campaigns regarding disasters, it is reported that there is still a significant lack of preparedness at the societal level (66, 69). Our study found that a significant proportion of parents do not engage in any disaster preparedness at home, which supports this situation (66). In contrast, the fact that parents who prepare at home have significantly higher DPS scores indicates that the strongest indicator of preparedness is direct behavior.

This finding suggests the necessity of interventions that include practical and family-specific planning rather than solely information-based approaches. It is critically important to personalize disaster plans for families with disabled members to include communication, evacuation, and continuity of care (22). At this point, psychological resilience can be considered an important resource that supports the capacity to sustain and implement the plan beyond initiating preparedness.

## 5 Conclusions and recommendations

The findings of this study indicate a small but statistically significant association between psychological resilience and disaster preparedness among parents of children with hearing impairments. Although psychological resilience appears to be related to preparedness, the magnitude of this association is modest, suggesting that disaster preparedness is influenced by multiple individual and contextual factors. Accordingly, psychological resilience should not be interpreted as a dominant driver of preparedness, but rather as one of several contributing factors within a broader, multifactorial framework. Given the cross-sectional design, causal relationships cannot be established, and resilience should be considered as one of several contributing factors rather than a primary determinant.

These findings may be interpreted within established theoretical frameworks. From a resilience theory perspective, the results suggest that resilience is not only an individual capacity but also a dynamic process that evolves in response to adversity. In line with ecological systems theory, disaster preparedness may be understood as an outcome shaped by the interaction between individual, family, and environmental factors. Additionally, from a family systems perspective, parental resilience may play a role in maintaining family functioning and supporting child safety during crisis situations. Therefore, this study contributes to the literature by highlighting the potential role of psychological resilience within a broader, multi-level context of disaster preparedness among families of children with hearing impairments. The findings also have implications for public health nursing practice. Public health nurses may play an important role in identifying vulnerable families, providing accessible disaster education, strengthening psychosocial resilience, and supporting communication-sensitive preparedness planning for families of children with hearing impairments. Community-based and family-centered nursing interventions may contribute to improving preparedness awareness and reducing disaster-related vulnerabilities among families with special needs.

Future research employing longitudinal or intervention-based designs is needed to better understand the direction and strength of this relationship. Practical implications should be considered as potential directions for future research and program development rather than direct conclusions drawn from the present findings.

In this context, a multidisciplinary perspective may be valuable in guiding future research and program development. Collaboration among audiologists, psychologists, educators, and local authorities may help address the diverse needs of families of children with hearing impairments. In addition, institutions responsible for disaster management, such as AFAD, may contribute to the development of accessible early warning systems, visual communication strategies, and inclusive preparedness initiatives. Such approaches may enhance the responsiveness of disaster preparedness efforts to both psychosocial and communication-related needs, particularly for families of children with hearing impairments.

## 6 Strengths and limitations of the study

This study contributes to the literature by examining the association between psychological resilience and disaster preparedness among parents of children with hearing impairments, a population that remains underrepresented in disaster and public health research. Given that most disaster preparedness studies focus on general population samples, addressing families of children with disabilities provides an important contextual contribution. Another strength of the study is the inclusion of both sociodemographic and disaster-related variables alongside psychological resilience within a hierarchical multiple regression framework, allowing a broader evaluation of factors associated with disaster preparedness. In addition, the study highlights the potential importance of accessible, family-centered, and disability-inclusive preparedness approaches in disaster-prone settings.

However, several limitations should be considered when interpreting the findings. First, the cross-sectional design precludes causal inferences and limits conclusions regarding the direction of the relationship between psychological resilience and disaster preparedness. Second, the use of self-report measures may have introduced response bias, including social desirability bias. Third, the study was conducted in a single province in Eastern Anatolia, which may limit the generalizability of the findings to different geographical, cultural, and institutional contexts.

Another important limitation is the absence of a comparison group consisting of parents of children without hearing impairments. Therefore, the findings cannot determine whether the observed preparedness patterns are specific to families of children with hearing impairments or reflect broader parental trends. Future studies including comparison groups may provide a more comprehensive understanding of disability-related and contextual influences on disaster preparedness.

Although hierarchical multiple regression analysis was conducted, some potentially relevant variables, particularly communication-related and caregiving factors, were not included in the model and may have influenced disaster preparedness behaviors. In addition, clinical characteristics of the children, such as the degree of hearing loss, type of assistive device used, and primary communication modality, were not assessed. These factors may influence communication barriers, caregiving demands, and preparedness experiences, and their absence may have limited the explanatory power of the model.

The fact that the Durbin–Watson statistic in the regression model falls below the ideal range suggests that the assumption of independent residuals may not have been fully met. Therefore, the final regression model was reanalyzed using HC3 robust standard errors, and the APRS total score remained significantly and positively associated with the total DPS score. However, due to the low Durbin–Watson value, the regression findings must be interpreted with caution.

The relatively wide age range of children (0–18 years) and the absence of developmental subgroup analyses may also limit the interpretation of age-specific caregiving and preparedness dynamics. Furthermore, the predominance of mothers in the sample should be considered, as mothers and fathers may differ in caregiving roles, preparedness perceptions, and coping strategies. Finally, some disaster-related variables, particularly household disaster preparedness status, may conceptually overlap with the outcome measure because they reflect related dimensions of preparedness behavior.

Future longitudinal, comparative, and intervention-based studies are needed to better clarify the contextual and multidimensional factors associated with disaster preparedness among families of children with hearing impairments.

## Data Availability

The raw data supporting the conclusions of this article will be made available by the authors, without undue reservation.

## Appendix 1 Sensitivity analysis using HC3 robust standard errors for Model 2

**Table:**

Independent variables		*B*	Robust SE	*t*	*p*	VIF
Age	25–31 years	2.772	2.251	1.232	0.220	3.444
	32–39 years	4.146	2.364	1.754	0.081	3.490
	≥40 years	1.046	2.583	0.405	0.686	3.183
Education	Secondary school	0.691	1.680	0.411	0.681	2.024
	High school	0.019	2.002	0.010	0.992	2.018
	University	−0.765	2.072	−0.369	0.712	2.309
Working status		1.286	1.539	0.836	0.404	1.341
Family type		−2.693	1.477	−1.823	0.070	1.148
Income status	Income equal to expenses	−0.678	1.219	−0.556	0.579	1.405
	Income greater than expenses	0.762	1.758	0.434	0.665	1.344
Consanguineous marriage status		−1.654	1.226	−1.349	0.179	1.461
Chronic disease in parent/spouse		0.486	1.468	0.331	0.741	1.250
All family members received sign language training		−0.897	1.071	−0.837	0.404	1.178
Previous disaster experience		2.500	1.181	2.117	**0.036**	1.147
Disaster-related education		3.121	1.403	2.225	**0.027**	1.283
APRS		0.158	0.070	2.248	**0.026**	1.504

HC3, heteroskedasticity-consistent type 3; *B*, unstandardized regression coefficient; robust SE, robust standard error; *t*, *t* statistic; VIF, variance inflation factor; DPS, Disaster Preparedness Scale; APRS, Adult Psychological Resilience Scale.
